# Neuroprotective Properties and Therapeutic Potential of Bone Marrow–Derived Microglia in Alzheimer’s Disease

**DOI:** 10.1177/1533317520927169

**Published:** 2020-06-15

**Authors:** Chang Li, Yu-Hua Chen, Ke Zhang

**Affiliations:** 1 Department of Developmental Cell Biology, Key Laboratory of Cell Biology, Ministry of Public Health, and Key Laboratory of Medical Cell Biology, Ministry of Education, China Medical University, Shenyang, China; 2 Department of Anesthesiology, Shengjing Hospital, China Medical University, Shenyang, China

**Keywords:** Alzheimer’s disease, microglia, bone marrow–derived microglia, amyloid beta, neuroprotection

## Abstract

Alzheimer’s disease (AD) is the most common form of dementia, which is characterized by a progressive cognitive decline and senile plaques formed by amyloid β (Aβ). Microglia are the immune cells of the central nervous system (CNS). Studies have proposed 2 types of microglia, namely, the resident microglia and bone marrow–derived microglia (BMDM). Recent studies suggested that BMDM, not the resident microglia, can phagocytose Aβ, which has a great therapeutic potential in AD. Bone marrow–derived microglia can populate the CNS in an efficient manner and their functions can be regulated by some genes. Thus, methods that increase their recruitment and phagocytosis could be used as a new tool that clears Aβ and ameliorates cognitive impairment. Herein, we review the neuroprotective functions of BMDM and their therapeutic potential in AD.

## Introduction

Alzheimer’s disease (AD) is the most common form of dementia worldwide, affecting 50 million people approximately.^1^ Majority of patients diagnosed as AD are 65 years or older.^2^ Every 5 years after the age of 65 years, the risk of developing the disease doubles, and above the age of 85 years, the risk reaches nearly 50%.^2^ Alzheimer’s disease is a heterogeneous neurodegenerative disease characterized by progressive dementia and the presence of plaques formed by amyloid β (Aβ).^3^ Inflammatory reaction around Aβ and accumulated proteins lead to senile plaques, which contain dystrophic neurites, activated microglia, and reactive astrocytes.^4^ Impaired Aβ clearance is one of the important mechanisms in AD. Microglia serve as a double-edged sword toward Aβ because they cannot only internalize and potentially degrade Aβ but also produce inflammatory mediators.^5^

Microglia are the immune cells in the central nervous system (CNS), constituting approximately 10% of the total glial cell population.^6^ They are located all over the brain and spinal cord, representing 5% to 15% of adult brain cells, with densities varying between distinct brain regions.^7^ The prevailing theory about the origin of microglia is that the precursor of hematopoiesis differentiates into microglia during embryogenesis.^8,9^ These precursors arise before E7.5 in mice and start to enter the CNS at E9 through the blood vasculature before the closure of the blood–brain barrier (BBB).^9,10^ In the CNS, microglia renew themselves via self-replication and/or progenitor cell division in the brain and maintain themselves via the proliferation of endogenous microglia in the normal mature brain.^11,12^ Another theory suggests that the microglia are produced postnatally from the engraftment of circulating monocytes called bone marrow–derived microglia (BMDM).^13^ The BBB prevent the peripheral blood cells infiltrating to CNS, and few peripheral cells are immersed in the brain in health. However, in pathological conditions, some CNS disorders, such as AD, peripheral monocytes, and macrophages, infiltrate into the brain with an accelerated process.^14^ The neuroprotective properties of BMDM in AD are highlighted.^15^

## Bone Marrow–Derived Microglia Have Neuroprotective Properties in AD

In the mouse model of AD, the beneficial effects of injection of umbilical cord blood-derived monocytes on the life duration were first proposed by Ende and colleagues.^16^ Since then, the effects of hematopoietic stem cell transplants such as cord blood or bone marrow grafts on cognitive function and amyloid deposition in AD have been extensively studied.^17^ Recent studies have shown that in the AD mouse model, after hippocampal injection of colony-stimulating factor 1, the bone marrow cells were stimulated to differentiate into BMDM-like cells expressing markers for microglia, including the recently identified transmembrane protein 119, which ameliorated the cognitive impairment in AD.^18^ These BMDM cells play important role in slowing the progression of AD, which may be mediated by the clearance of Aβ.

### Bone Marrow–Derived Cells Engraft the CNS in AD

A great progress of migration of bone marrow–derived cells into the CNS in AD has been made after the establishment of the bone marrow chimeric mouse model, allowing the blood cell–specific detection in the brain, which expresses green fluorescent protein (GFP).^19^ The enhanced green fluorescent protein (eGFP) expressing bone marrow–derived cells were transplanted into 2.5-month-old APP/PS1 mice, and results showed that the density of bone marrow–derived eGFP-positive cells in the brain was significantly higher in mice with AD than in transplanted wild-type mice at the age of 9 months, which indicates a higher infiltration activity of BMDM in mice with AD.^20^ Simard and colleagues have carried out a detailed study to confirm whether the infiltration of BMDM was specific to Aβ.^21^ They injected synthetic Aβ (1-40) and Aβ (1-42) into the hippocampus, which led to an increased infiltration of BMDM in the brain 72 hours after injection.^21^

**Figure 1.**
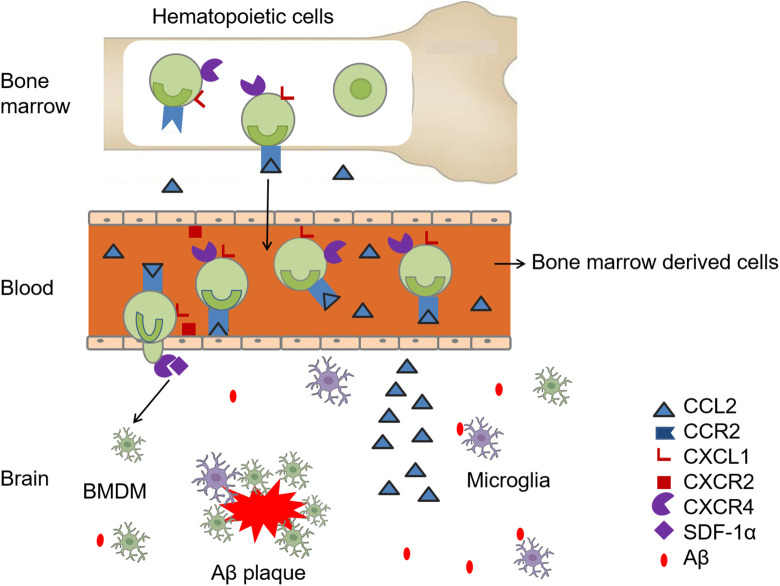
A multistep process of bone marrow–derived microglia (BMDM) recruitment into brain in Alzheimer’s disease (AD). With stimulation of amyloid β (Aβ), the recruitment of BMDM is enhanced. CC-chemokine ligand 2 (CCL2) is produced by brain cells. CC-chemokine receptor 2 (CCR2)/CCL2 is necessary for cells engraftment from bone marrow into blood. Chemokine (C-X-Cmotif) ligand 1 (CXCL1), after binding with CXC chemokine receptor 2 (CXCR2) in human brain microvascular endothelial cells, derives transendothelial migration of bone morrow–derived cells and stromal-derived factor 1 (SDF-1)/ CXC chemokine receptor 4 (CXCR4) controls the trafficking of monocytes into the brain parenchyma.

The engraftment of bone marrow–derived cells into the CNS and the migration to Aβ deposition is a multistep process. CC-chemokine receptor 2 (CCR2) is one of the chemokine receptors that bind to CC-chemokine ligand 2 (CCL2), which is essential in the process of monocyte mobilizing from the bone marrow and engrafting to inflammatory sites in AD.^22^ When Tg2576 mice with AD were crossed with (CCR2^−/−^) mice, blood-derived monocytic cells were fewer, indicating that the CCR2-dependent infiltration of blood-derived monocytic cells may exist in AD.^23^ Recent studies have demonstrated that the CCL2/CCR2 pathway affects the migration of monocytes in some inflammation models such as sterile peritonitis, atherosclerosis, and Listeria infection,^24^ suggesting that the same process may also exist in the brain of a patient with AD. Research showed that chemokine (C-X-C motif) ligand 1 facilitates the Aβ-induced transendothelial migration via the endothelial tight junction, which is overexpressed by monocytes, derived from patients with AD and interacting with CXC chemokine receptor 2 (CXCR2) in human brain microvascular endothelial cells.^25^ The CX3CL1/CX3CR1 pathway was involved in the adhesion of monocyte at the blood vessel to tissue,^26^ and stromal-derived factor 1α (SDF-1α) controls the trafficking of monocytes into the brain parenchyma by conjunction with its receptor CXCR4.^27^ This leads to the hypothesis that the migration of monocytes from the blood to plaque in brain is a multistep process, controlled by distinct chemokine receptor pairs that act at different process (Figure 1).

There are other factors involved in the migration of BMDM. CCL5, secreted from the transplanted bone marrow–derived mesenchymal stem cells, drives the recruitment of the alternative microglia into the brain, which is induced by Aβ deposition in the AD brain.^28^ Very late antigen 4 (VLA-4) expressed by monocytes participates in monocyte migration into the CNS through binding vascular cell adhesion molecule 1 (VCAM-1) upregulated on endothelium in several models of inflammation such as encephalomyelitis and spinal cord injury.^29,30^ The VLA-4 antibody suppresses the recruitment of monocytes to the infected brain.^31,32^ Similar functions are observed between lymphocyte function-associated antigen 1 (LFA-1), which are expressed by monocytes, and their ligands intercellular adhesion molecule-1 (ICAM-1) on endothelium,^32^ and these results remind us that VLA-4, VCAM-1, LFA-1, and ICAM-1 may also have a role in recruitment of monocytes to brain in AD.

### Bone Marrow–Derived Microglia Are Effective in Aβ Clearance and Ameliorating Cognitive Dysfunction

Previous study showed that after 6 months of bone marrow transplantation, the number and the size of amyloid plaques in APP_Swe_/PS1 mice brain were reduced obviously.^21^ Bone marrow–derived microglia are very efficient in restricting amyloid deposits. Bone marrow–derived microglia secrete proteins that promote degradation and phagocytosis of Aβ, which may be more prominent than that of the resident microglia.^33^ Bone marrow–derived microglia migrate to amyloid plaques and clear Aβ species via endocytosis.^21^ CCR2^+^ bone marrow cells are thought to be largely responsible for the blood-derived microglia in AD. APP_Swe_CCR2^−/−^ mice show higher Aβ deposition around blood vessels and rapid cognitive decline than APP_Swe_CCR2^+^^/^^+^, which suggests that CCR2 deficiency significantly impaired Aβ clearance and amplified vascular Aβ deposition.^34^ By contrast, memory capacities could be restored after transplanting of wild-type bone marrow stem cells.^35^ This suggests that the clearing of Aβ promoted by BMDM may occur in a CCR2-dependent manner.

Toll-like receptors (TLRs) are involved in Aβ phagocytosis. Mononuclear cells of patients with AD fail to upregulate TLRs with Aβ stimulation, in contrast to those from normal subjects that upregulate TLR expression.^36^ Delivering of TLR2-lentiviral genes into bone marrow–derived cells can rescue cognitive decline in TLR2-deficient AD mice.^37^ Normal human monocyte TLR4 levels may be higher than that in patients with AD. Bisdemethoxycurcumin is an anti-inflammatory compound, which can promote the clearance of Aβ and regulate the expression of TLR2-4 in monocytes.^36^ These studies highlight the function of TLRs in peripheral blood mononuclear cells (PBMCs) in AD.

Besides phagocytosis, Aβ may be cleared by cerebral infiltrating monocytes through the secretion of proteolytic enzymes.^38^ Studies have shown that these glatiramer acetate-treated macrophages release matrix metalloproteinase 9 to degrade Aβ.^38,39^ The ganciclovir was injected into the mice which is the offspring of the APP_Swe_/PS1 mice and the transgenic mice expressing the thymidine kinase protein to eliminate the BMDM in the brain.^20^ Four weeks after injection, these mice had higher Aβ plaques and amyloid plaques, suggesting that BMDM is essential for Aβ clearance.^20^

### Gene Associated With the Function of BMDM

The expression of β-1,4-mannosyl-glycoprotein 4-β-N-acetylglucosaminyl transferase (MGAT3) was decreased in PBMCs in patients with AD, whereas the opposite was observed in control patients exposed to Aβ.^36^ Using MGAT3, small interfering RNA to silence MGAT3 expression inhibited the uptake of Aβ to PBMCs isolated from healthy patients and inhibited monocyte clustering around Aβ, suggesting that MGAT3 may be important in the uptake and degradation of Aβ.^36^

Apolipoprotein E4 (APOE4) genotype is the strongest genetic risk factor for late-onset AD.^40^ Bone marrow transplant (BMT) was performed from GFP expressing human APOE3/3 or APOE4/4 donor mice into APP_Swe_/PS1DE9 mice.^41^ APOE4/4 recipient APP_Swe_/PS1DE9 mice demonstrated significantly impaired spatial working memory, increased level of Aβ, and reduced BMT-derived microglia engraftment.^41^ While APOE3/3 BMT recipients express less tumor necrosis factor α, macrophage migration inhibitory factor and more immunomodulatory IL-10 expression comparied with APOE4/4 BMT recipients.^41^ These findings suggest that BMT-derived APOE3-expressing cells are superior to those that express APOE4 in reducing the behavioral and neuropathological changes in experimental AD.^41^ Macrophages from ApoE2 mice are more efficient in degrading Aβ than those from ApoE3, which in turn are better phagocytes than the macrophages from ApoE4.^39^

ATP-binding cassette transporter A7 (ABCA7) is a genetic risk factor for late-onset AD.^42^ Researchers crossed ABCA7-deficient mice with J20 amyloidogenic mice, through which they discovered ABCA7 loss doubled insoluble Aβ levels in the brain.^43^ Their study showed that bone marrow–derived macrophages derived from ABCA7-deficient mice had a 51% reduction in the ability to take up oligomeric Aβ compared to wild-type mice.^43^

Bone marrow cells derived from mice homozygous deficient for prostaglandin E2 receptor subtype 2 (EP2) was transplanted into APP_Swe_-PS1dE9 double transgenic AD mouse.^44^ A 25% reduction in cerebral cortical Aβ burden was achieved after BMT, which provided a foundation for transplantation with EP2-null bone marrow in suppressing accumulation of Aβ peptides^44^ (Table 1).

**Table 1.** Gene Associated with the Function of BMDM.

Study	Gene	Model	Effect on Aβ burden
Fiala et al^36^	MGAT3	MGAT3 siRNA to inhibition MGAT3 expression in PBMCs	MGAT3 is important in the uptake and degradation of Aβ
Yang et al^41^	APOE3/3 APOE4/4	BMT from green fluorescent protein expressing human APOE3/3 or APOE4/4 donor mice into APP_Swe_/PS1DE9 mice	APOE3-expressing cells are superior to those that express APOE4 in their ability to clear Aβ
Kim et al^43^	ABCA7	Crossed ABCA7-deficient mice with J20 amyloidogenic mice	Absence of ABCA7 leads to reduced take-up of oligomeric Aβ
Keene et al^44^	EP2	EP2^−/−^ bone marrow–derived cell chimera in APP_Swe_/PS1dE9 mice	EP2 deletion in BM cells reduces Aβ burden
Mildner et al^34^	CCR2	CCR2^−/−^ BM cell chimera in APP_Swe_/PS1 and Tg2576 mice	CCR2 expression in BM cells are required for their brain engraftment. Peripheral macrophages rather than parenchymal microglia modulate Aβ deposition in AD mice
Town et al^45^	TGF-β	Dominant negative TGF-β in CD11c^+^ cells in Tg2576 and APP_Swe_/PS1dE9 mice	TGF-β deficiency in CD11c^+^ reduced Aβ burden involving infiltration of peripheral macrophages
Hao et al^46^	Myeloid differentiation factor 88	MyD88^−/−^ BM cell chimera in TgCRND8 and APP_Swe_/PS1dE9 mice	MyD88 deletion in BM cells attenuates neuroinflammation, enhances Aβ phagocytosis, and reduces Aβ burden

Abbreviations: Aβ, amyloid β; AD, Alzheimer’s disease; BM, bone marrow; BMT, bone marrow transplant; CCR2, CC-chemokine receptor 2; PBMCs, peripheral blood mononuclear cells; TGF-β, transforming growth factor β.

## Therapeutic Potential of BMDM in AD

### Using Hematopoietic Cell Transplantation in the Turnover of Brain Microglia

The resident microglia and microglia-like cells have similar features such as ramified morphology and characteristic pattern of ion channels.^47^ When neuronal damage occurs, these cells could migrate and send processes in a way similar to the microglia.^48^ Overall, the newly engrafted myeloid cells (particularly BMDM cells) played a role in surveillance and scavenging functions similar to those of the microglia, which made them a potential target in the treatment of AD. Juzen-Taiho-To, a herbal medicine, promotes the differentiation of transplanted bone marrow cells into the microglia and phagocytosis of Aβ, which contributes to the reduction of Aβ burden.^49^ This suggests that stimulating recruitment of BMDM may be an effective way to treat AD because of the neuroprotective effects of BMDM.

Hematopoietic cell transplantation (HCT) can stimulate recruitment of BMDM to the brain; however, the process of turning over the brain microglia after HCT is extremely slow. Twelve months after transplantation, the rate of donor-derived microglia engraftment was approximately 25%.^50^ A more efficient turnover of microglia was achieved through the busulfan conditioning regimen, which depleted endogenous microglia and created space for the engraftment.^51^ The proliferation of donor-derived cells and the appearance of more mature donor cells indicate amplification and maturation of donor-derived precursor cells, which can migrate into the brain and proliferate locally, led to the myelomonocytic reconstitution after myeloablation.^52^ These data have presented a process of reconstitution of differentiated, resting microglia after HCT. In latest study, HCT from bone marrow moved into peripheral blood to differentiate into microglia-like cells which expressed microglial markers and engaged in Aβ phagocytosis. These peripheral blood-derived microglia-like cells can be obtained directly and much more safer than traditional HTC. It is a hopeful source for a novel cell therapy against AD especially for elder people.^53^

### Enhance the Migration of BMDM to Brain

Stromal-derived factor 1α, an effective chemoattractant for hematopoietic progenitor cells, might participate in the migration of BMDM from peripheral cycle to brain, therefore providing a promising target for the treatment of AD.^54^ A research was conducted to explore the function of the combined use of granulocyte colony-stimulating factor (G-CSF), AMD3100 (CXCR4 antagonist), and SDF-1α. The results showed that SDF-1α was effective in inducing migration of the endogenous bone marrow–derived hematopoietic progenitor cells into brain, mobilized by G-CSF/AMD3100 which acts synergistically to produce a therapeutic effect.^55^ Other approaches to increase the delivery of bone marrow–derived cells to the brain include intranasal application, which has a higher efficiency to deliver bone marrow–derived cells to the brain.^56^

### Promote the Ability of BMDM to Clear Aβ and Reduce Inflammatory

**Figure 2.**
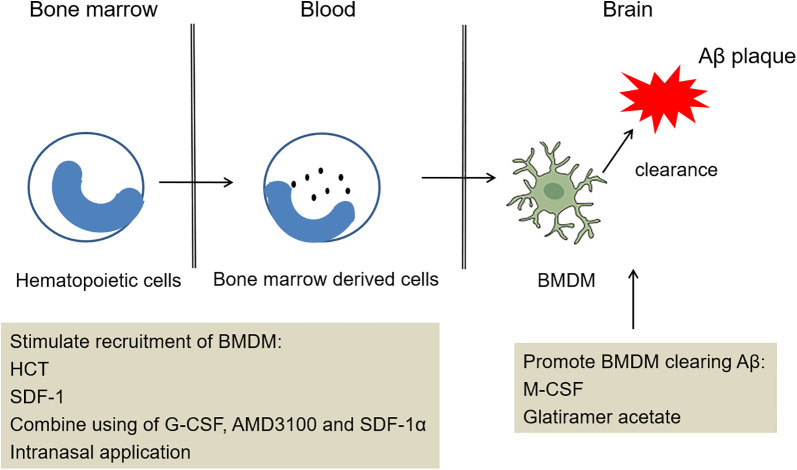
Potential treatment strategy of bone marrow–derived microglia (BMDM) recruitment into the brain. The treatment acts on different stages in the pathogenesis of Alzheimer’s disease (AD). This treatment clears amyloid β (Aβ) ultimately involved stimulating recruitment of BMDM, promoting BMDM clearing Aβ.

Recent studies demonstrated the beneficial effects of macrophage colony-stimulating factor (M-CSF) in AD.^57^ Macrophage colony-stimulating factor injected on a weekly basis prior to the appearance of learning and memory deficits showed an increased number of microglia in the parenchyma, decreased number of Aβ deposits, and less Aβ40 and Aβ42 monomers in the extracellular protein, which suggested M-CSF can stimulate BMDM, degrade Aβ, and have protective effects in AD.^57^ Glatiramer acetate and minocycline may have protective effects in AD through the enhancement of the activity of the bone marrow monocyte-derived macrophages and reduce overall inflammatory potential of bone marrow monocyte–derived cells, respectively^38,58^ (Figure 2).

## Conclusion

A deeper exploration for the function and mechanism of BMDM in the development of AD is needed before therapeutic strategies can be developed. The infiltration of BMDM cells may be a good therapeutic approach since these cells have a high capacity to phagocytose amyloid. In addition, the self-donation of bone marrow or peripheral blood is safe, widely applicable, and ethically acceptable. Overall, the BMDM cells have the potential as a cell-based disease-modifying therapy against AD.
